# I'd Do Anything for Research, But I Won't Do That: Interest in Pharmacological Interventions in Older Adults Enrolled in a Longitudinal Aging Study

**DOI:** 10.1371/journal.pone.0159664

**Published:** 2016-07-20

**Authors:** Matthew Calamia, John P. K. Bernstein, Jeffrey N. Keller

**Affiliations:** 1 Department of Psychology, Louisiana State University, Baton Rouge, Louisiana, United States of America; 2 Pennington Biomedical Research Center, Institute for Dementia Research and Prevention, Baton Rouge, Louisiana, United States of America; Banner Alzheimer's Institute, UNITED STATES

## Abstract

Alzheimer’s disease (AD) ranks as the 6^th^ leading cause of death in the United States, yet unlike other diseases in this category, there are no disease-modifying medications for AD. Currently there is significant interest in exploring the benefits of pharmacological treatment before the onset of dementia (e.g., in those with mild cognitive impairment); however, recruitment for such studies is challenging. The current study examined interest in pharmacological intervention trials relative to other types of clinical interventions. A total of 67 non-demented older adults enrolled in a longitudinal cognitive aging study completed a questionnaire assessing interest in participating in a variety of hypothetical research study designs. Consistent with past research, results showed that the opportunities for participants to advance science, receive feedback about their current health, and help themselves or others, were associated with increased interest in clinical trial participation. Some factors were not associated with change in interest (e.g., a doctor not recommending participation) while others were associated with decreased interest (e.g., having to come in for multiple visits each week). Relative to other types of interventions, pharmacological intervention trials were associated with the least interest in participation, despite pharmacological interventions being rated as more likely to result in AD treatment. Decreased interest was not predicted by subjective memory concerns, number of current medications, cardiovascular risk, or beliefs about the likely success of pharmacological treatments. These results highlight the challenges faced by researchers investigating pharmacological treatments in non-demented older individuals, and suggest future research could contribute to more effective ways of recruiting participants in AD-related clinical trials.

## Introduction

Alzheimer’s Disease (AD) is currently the sixth-leading cause of death in the United States, and is the only disease in the top ten for which there are no disease-modifying medications. The Collaboration for Alzheimer’s Prevention and other organizations have highlighted that doing clinical trials in individuals with mild cognitive impairment (MCI), or participants with no cognitive impairment but at increased risk for AD (e.g., genetic factors, potential AD biomarkers, etc.) is essential for the development of interventions that are effective in the eventual treatment of AD [1]. There are approximately 300 clinical trials underway, or soon to be underway, in the United States that are designed to determine the ability of different interventions to prevent or delay the development of AD [2]. Some of these strategies involve pharmacological interventions, while others involve non-pharmacological approaches including environmental or behavioral based interventions [3], [4]. Currently, it is unknown what motivates or deters an individual from participating in different types of clinical trials related to AD. Understanding the factors that influence, or predict, the willingness of an individual to participate in the different types of interventions is important for developing the most effective recruitment strategies for a specific type of clinical trial [1], [5].

Pharmacological interventions are one type of intervention with some success in dementia-related clinical trials; SomeScurrent pharmacological interventions slow cognitive decline in individuals at risk of developing AD [6–8]. Unfortunately, research on these interventions is limited by a number of obstacles, including the limited availability of individuals willing to participate in dementia-related clinical trials [9]. A large number of issues including medical comorbidities and lack of a widespread registry of potential clinical trial participants hampers recruitment into dementia-related trials [10]. Other factors such as the use of concomitant medications, and the lack of an adequate informant or study partner to complete study measures also lead to exclusion from dementia-related clinical trials [11]. This dearth of eligible participants may contribute to prolonged periods of recruitment for Phase II and Phase III multisite dementia-related drug trials; one study found the median time for recruitment across 29 dementia-related studies was 17 months [12].

Among individuals who are qualified to participate, many still fail to enroll for a variety of reasons including a lack of interest and logistical issues associated with participation (e.g., time, travel). In a large, multi-center prevention study using a supplement (Ginkgo Biloba), investigators aimed to recruit individuals with normal cognition or mild cognitive impairment (MCI) and found that a lack of interest was the primary reason individuals declined to participate [13]. Research using focus groups has shown that logistical issues such as difficulty arranging transportation and distance to research facilities are frequently cited as a barrier to participation in dementia research in general [14], [15].

Individuals may also be disinclined to enroll in clinical drug trials because they are averse to the potential risks associated with drug treatment; when presented with hypothetical scenarios, nearly half individuals gave a response indicating fear associated with taking a drug for research purposes [16]. Compared to other types of research studies, individuals often indicate less interest in enrolling in clinical drug trials. For example, when presented with hypothetical research scenarios, caregivers of AD patients were more likely to express interest in having the patient enroll in a study that involved neuroimaging and neuropsychological tests as compared to a study that included the use of an experimental drug [17]. In another study using hypothetical scenarios, AD patients reported a greater willingness to take part in a hypothetical blood draw study than a drug study [18].

Aversion to drug studies may reflect a broader aversion to risk-taking that is typical in older individuals [19]. While those with MCI may underestimate risks and be more willing to enroll in trials [20], AD patients may be more risk adverse than cognitively healthy individuals [18].

Participants and caregivers who participate in AD research, including clinical drug trials, most commonly cite the potential for direct benefits and a desire to help others as reasons for participation [21–23]. Among those already enrolled in an AD research registry, more favorable attitudes toward research was associated with a larger number of positive responses to being approached about research studies [24]. More favorable attitudes towards research also predicted a greater positive response to enrollment in a hypothetical clinical drug trial [25].

Given the promise that pharmaceuticals hold with regard to improving cognitive outcomes in dementia populations, there is a substantial interest in determining how to increase enrollment in dementia-related clinical trials. The aims of this study were: 1) to compare participant interest in pharmacological interventions to their interest in other types of research studies varying in benefits, requirements, and intervention type, 2) to compare beliefs about likely success of pharmacological interventions to other types of interventions, and 3) to identify individual difference factors associated with a lack of interest in participating in clinical drug trials. To extend existing research, we focused on variables which have been less explored as predictors of interest, including health, subjective memory concerns, and beliefs about the likely success of future drug treatments.

## Methods

### Participants

Participants were recruited from Louisiana Aging Brain Study (LABrainS), a longitudinal study of cognitive aging conducted by the Institute for Dementia Research and Prevention (IDRP) at the Pennington Biomedical Research Center. This is an active study in which individuals volunteer to receive annual cognitive and mobility evaluations and complete other ancillary studies in order to examine the relationship of various factors to longitudinal changes in cognition and mobility and to aid in the development and refinement of clinical and research measures [26–31]. Participants in the LABrainS study are non-demented older adults recruited through outreach efforts of the IDRP throughout Louisiana and surrounding states. A total of 60 randomly selected healthy elderly and 60 randomly selected MCI participants were invited by email solicitation to participate, of which 67 (56%) responded and were included in analyses. Participants were identified as being healthy elderly or MCI participants based on their most recent LABrainS visit (i.e. within the past twelve months). Participants sent their responses by mail and received $20 upon return of the questionnaire. Written informed consent was obtained from participants and the study was approved by the Pennington Biomedical Institutional Review Board and Ethics Committee.

Participants were 64.2% female (n = 43) and had an average age of 70.4 (*SD* = 5.8) years (range = 55–85). 68.7% (n = 46) of participants held a college degree, while 23.9% (n = 16) had completed some college and 7.5% (n = 5) had a high school diploma. All participants were white. 76.1% (n = 51) of participants were married, 11.9% (n = 8) were divorced, 6% (n = 4) never married and 6% (n = 4) were widowed. 64.1% (n = 43) of participants were cognitively healthy and 35.8% (n = 24) had MCI. Determination of MCI status was based on performance on the Repeatable Battery for the Assessment of Neuropsychological Status (RBANS) [32], a brief neuropsychological battery which has been shown to be sensitive to MCI [33].

### Measures

A survey was designed assessing participants’ current health, concerns about memory problems, interest in participating in studies with varying characteristics (e.g., type of intervention, time commitment), and beliefs about the likelihood that different types of interventions will lead to a successful treatment for chronic neurological diseases such as AD. Health was assessed by questions asking about the presence or absence of a specific condition (i.e., diabetes, high blood pressure, cardiovascular disease, cancer, stroke, Parkinson’s disease). Two or fewer participants endorsed the last three conditions (i.e., cancer, stroke, Parkinson’s disease) and those conditions were not analyzed further. Subjective memory concerns were also measured with one dichotomous item (i.e., “Are you concerned about your memory?”). To measure interest in studies with varying characteristics, participants were asked to describe the degree to which each of 29 study characteristics would impact their decision to participate in clinical research. Following the presentation of a single, specific feature (e.g., if the study involved a dietary intervention), participants chose one of three responses to indicate the effect of this feature on their interest in participation: “This would significantly increase the chances I would participate”, “This is not a major factor in my deciding to participate”, or “This would significantly decrease the chances that I would participate”. To measure beliefs about the success of various treatments, participants were asked to indicate how likely each of 9 interventions would lead to a treatment for neurological diseases such as AD. Participants responded on a four-choice scale (“Not likely”, “Possible”, “Likely” or “Highly Likely”).

### Analyses

Statistical analyses were conducted using IBM SPSS Statistics (Version 22). Percentages reflecting increases and decreases in participation related to specific hypothetical study characteristics were calculated to illustrate the relative ranking of interest in pharmacological trials. McNemar’s Test was used to compare interest in participation in a pharmacological trial to interest in participation in other types of interventions. McNemar’s Test was also used to compare beliefs about the likelihood a pharmacological intervention would lead to a successful treatment to beliefs about other types of interventions. Logistic regression was used to determine if decreased interest in participating in a pharmacological trial could be predicted by: how likely the participant thinks a drug will treat a chronic neurological disease such as AD, whether or not the participant is currently concerned about his or her own memory, the current number of medications the participant is taking, and cardiovascular risk (a unit-weighted score based on the presence of diabetes, high blood pressure, a sedentary lifestyle, obesity, and high cholesterol).

## Results

### Pharmacological Intervention vs. Other Study Characteristics

Table 1 shows the percentages of participants indicating increased interest, no change in interest, or decreased interest given the presence of various study characteristics. Potential benefits for self and others were generally strongly associated with increased interest in participation. A large number of participants (52%) indicated decreased interest if the study was a pharmacological trial. The only study characteristics associated with greater decreased interest in participation were having to receive a lumbar puncture (68%) or having to come in for study visits three times a week (73%) or daily (86%).

**Table 1:** Research Designs and Interest in Study Participation.

	Significant increase likelihood of participation N (%)	Not a major factor in decision to participate N (%)	Significantly decrease likelihood of participation N(%)
**Benefits for participant**			
Study topic interests me	61(92.4)	5(7.6)	0 (0%)
Researcher contacts me	41(62.1)	25(37.9)	0 (0%)
Help my health	65(98.5)	1(1.5)	0 (0%)
Get feedback on my health	62(93.9)	4(6.1)	0 (0%)
Receive payment	11(16.4)	54(83.1)	0 (0%)
**Benefits for others**			
Leads to treatment for disease	63(95.5)	3(4.5)	0 (0%)
Advances science	59(89.4)	7(10.6)	0 (0%)
Help others	62(93.9)	4(6.1)	0 (0%)
**Medical procedure**			
Have MRI	10(15.6)	47(73.4)	7(10.9)
Have lumbar puncture (spinal tap)	2(3.1)	19(29.2)	44(67.7)
Provide blood sample	10(15.4)	54(83.1)	1(1.5)
**Types of interventions**			
Diet	6(9.8)	40(65.6)	15(24.6)
Medication	4(6.2)	27(41.5)	34(52.3)
Exercise	30(45.5)	36(54.5)	0 (0%)
Meditation	13(19.7)	40(60.6)	13(19.7)
Acupuncture	13(19.7)	33(50)	20(30.3)
Yoga	14(21.5)	30(46.2)	21(32.3)
Computer-based	24(36.9)	37(56.9)	4(6.2)
**Intervention Characteristics**			
1 month long	24(36.9)	41(63.1)	0 (0%)
3 months long	12(18.5)	49(75.4)	4(6.2)
6 months long	9(13.6)	51(77.3)	6(9.1)
12 months long	8(12.7)	46(73)	9(14.3)
1 onsite visit each week	9(13.6)	43(65.2)	14(21.2)
3 onsite visits each week	3(4.5)	15(22.7)	48(72.7)
Daily onsite visits each week	0 (0%)	9(13.6)	57(86.4)
Might be in control group	5(7.6)	47(71.2)	14(21.2)
**Recommendations of PCP**			
Doctor recommends I participate	58(87.9)	8(12.1)	0 (0%)
Doctor doesn't recommend I participate	5(7.8)	39(60.9)	20(31.3)

### Pharmacological Intervention vs. Other Interventions

Given the small number of participants indicating an increased interest in a pharmacological intervention (n = 4), the categories of increased interest and no change in interest were collapsed to allow for statistical comparisons with other interventions. Of all the interventions studied, pharmacological interventions were associated with the greatest amount of decreases in interest in participation. This difference was significant for all comparisons of interest in participation in a pharmacological intervention to interest in participation in other interventions (McNemar’s Test, all p <.05).

In contrast to the results for interest in participation, pharmacological interventions were seen as more likely to lead to a treatment for chronic neurological diseases like AD than several other interventions. 71% of participants rated pharmacological interventions as “likely” or “highly likely” to lead to a treatment. This percentage was higher than the rating for meditation (26%, p <.01) acupuncture (23%, p <.01), yoga (29%, p <.01) and computer-based interventions (51%, p <.05), but not exercise (78%, p = .56) or dietary interventions (65%, p = 0.52).

### Predictors of Decreased Interest in Participating in a Pharmacological Intervention Trial

Compared to the collapsed categories of increased interest or no change in interest, decreased interest in participation could not be significantly predicted by a model including belief that drug treatments would lead to cures for diseases like AD, current memory concerns, the number of current medications taken, or cardiovascular risk (χ2 (4) = 4.20, p = 0.38). Given the number of participants, the planned simultaneous analysis of multiple predictor variables was followed-up by a post-hoc examination of bivariate correlations of each predictor with interest in participation. No significant correlations were found (p>0.05). In response to a reviewer’s feedback, an additional post-hoc analysis of age, gender, and education was conducted; this demographic model did not significantly predict interest in participation (χ2 (4) = 1.98, p = 0.74).

## Discussion

Given the number of pharmacological interventions planned or underway for delaying or preventing the onset or progression of AD, and the challenges in recruitment for those studies, understanding factors that increase or decrease enrollment in research is an important goal for clinical trial researchers. Some barriers to enrollment cannot be solved by increasing interest when potential participants are identified (e.g., exclusions from enrollment due to medical comorbidities or stage of disease). However, other barriers are related to an individual’s interest in a study and decision to enroll or decline participation. For example, fewer positive attitudes towards research and an aversion to drug-related side effects have been associated with disinterest in clinical trial participation [11], [13], [21–23]. In this study, we explored how interest in pharmaceutical trials compared with interest in clinical research studies with different characteristics, including studies using other types of interventions. We also explored whether individual differences in health, subjective memory concerns, and beliefs about the likely success of pharmacological interventions were related to interest in participation. Participants were those already enrolled in a longitudinal study of aging, an important source of potential recruitment into intervention studies given the emphasis of many clinical trials to recruit healthy individuals or individuals with mild cognitive impairment into interventions designed to delay potential future pathological changes.

The current study showed that individuals were more likely to enroll in research studies when they believed that their participation would help improve the health of others and themselves, as well as learn about their health. These findings are congruent with those of prior studies examining the decision-making of both AD patients and caregivers, two of which found that the main reasons patients joined a dementia registry, were to help others (44% of patients) and themselves (29%) [21], [23]. Similar to previous work, individuals in the current study were less likely to enroll if participation would require significant amounts of time and traveling (i.e., traveling to the study site several times per week) [14], [34].

The type of intervention involved played a significant role in individuals’ interest in participation. Although 95% of participants reported that they would be more likely to participate if a study led to a treatment for a disease, the type of intervention still mattered. Interest in a pharmacological trial (48%) was lower than interest in all other types of interventions: dietary, exercise, meditation, acupuncture, yoga and computer-based interventions. Of note, some of these other interventions were rated as less likely to lead to a treatment for diseases like AD compared to pharmacological interventions. A total of 71% of participants viewed pharmacological interventions as being likely or highly likely to lead to a treatment compared to interventions such as meditation, acupuncture, yoga, and computer-based interventions.

Beliefs that drug treatments would lead to cures for diseases, current memory concerns, the number of current medications taken, and cardiovascular risk did not predict the participants’ interest in clinical trial participation. These results extend prior work that found that subjective memory and cardiovascular disease did not predict willingness to participate in AD research more generally [35]. Future work is aimed at identifying individual difference factors related to interest in various types of clinical trials.

Although sample size is a limitation, the number of participants in this study is comparable to previous studies using a similar survey-based approach to assessing older adults’ interest in research [16], [24]. We conducted a logistic regression with four predictor variables and 34 events for 65 participations. In order to conduct a logistic regression, ten events per predictor variable is a common rule of thumb for sample size [36]; however, others have argued that this may be too strict, with simulations showing that as few as five events per predictor may be acceptable [37]. It should be noted that majority of variables were measured using a single item which may have limited reliability. This format was chosen to allow for the inclusion of a number of study features which could be asked about independently; future studies may wish to use more realistic scenarios in which participants are provided with more details about specific studies to gauge their interest in participation.

It should be noted that only 36% of the final sample were individuals with MCI, limiting our generalizability to that population. We did not directly compare individuals with and without MCI in this manuscript as that categorization overlaps significantly (although not entirely) with the subjective memory complaints variable we used as a predictor. At least one previous study did find a difference between those with MCI and cognitive healthy older adults in their interest in participation in a drug trial presented in a hypothetical vignette [38]. As both of these groups are target populations for clinical trials, future studies should continue to explore possible differences between these groups in factors affecting their interest in trial participation.

Another limitation of this study was that participants were not responding to questions about actual available clinical trials. Throughout completion of the questionnaires, participants were aware that they were expressing interest in participating in studies that were not currently being held at the IDRP, and thus their responses had no bearing on their actual enrollment in any studies. This may have led to an overestimation of the actual number of participations who would have participated in an actual trial. Previous work in another domain (HIV prevention) indicates that participants are more likely to report a willingness to enroll in medication intervention trials when the study is presumed to be hypothetical [39].

The use of hypothetical studies in survey research is common in the clinical trial literature for MCI/AD; few studies have experimentally manipulated variables to observe effects on trial enrollment [40]. Those that have done so have limited their focus to factors such as the type of study advertisement (direct mailings vs. newspaper advertisements vs. community outreach [41] or the party to which advertisement efforts were targeted (primary care provider vs. larger community [42]. To our knowledge, the effect of manipulations in how study information is presented to participants on recruitment has not been studied in actual pharmacological treatment trials.

Given research showing that fear of potential side effects is one barrier to participation in pharmacological treatment trials [16], to the extent such perceptions are inaccurate or fail to consider potential benefits, future research could investigate the effect of manipulations in how information on risk and benefit is presented to potential participants in clinical trials. In the larger literature on medical decision-making, a number of communication strategies have been successfully used to improve understanding of risks and benefits [43]. Given individuals’ willingness to be contacted about enrolling in future AD studies has shown susceptibility to change over the time course of participation in a longitudinal study [44], researchers should continue to explore ways to increase enrollment in pharmacological intervention trials in individuals already enrolled in a research registry.
